# A simple method to measure CLOCK-BMAL1 DNA binding activity in tissue and cell extracts

**DOI:** 10.12688/f1000research.11685.2

**Published:** 2017-09-12

**Authors:** Maud Gillessen, Pieter Bas Kwak, Alfred Tamayo

**Affiliations:** 1 Department of Neurobiology, Harvard Medical School, Boston, MA, 02115, USA; 2 Department of Biology, University of Namur, 5000 Namur, Belgium

**Keywords:** circadian clock, DNA binding assay, CLOCK, BMAL1, phosphorylation, chromatin

## Abstract

The proteins CLOCK and BMAL1 form a heterodimeric transcription factor essential to circadian rhythms in mammals.  Daily rhythms of CLOCK-BMAL1 DNA binding activity are known to oscillate with target gene expression in vivo. Here we present a highly sensitive assay that recapitulates native CLOCK-BMAL1 DNA binding rhythms from crude tissue extracts, which we call the Clock Protein-DNA Binding Assay (CPDBA). This method can detect less than 2-fold differences in DNA binding activity, and can deliver results in two hours or less using 10 microliters (~10 micrograms) or less of crude extract, while requiring neither specialized equipment nor expensive probes. To demonstrate the sensitivity and versatility of this assay, we show that enzymatic removal of phosphate groups from proteins in tissue extracts or pharmacological inhibition of casein kinase I in cell culture increased CLOCK-BMAL1 DNA binding activity by ~1.5 to ~2 fold, as measured by the CPDBA. In addition, we show that the CPDBA can measure CLOCK-BMAL1 binding to reconstituted chromatin. The CPDBA is a sensitive, fast, efficient and versatile probe of clock function.

## Introduction

The maintenance or disruption of circadian rhythms contribute significantly to several areas of health and disease ( Asher & Schibler, 2011; Chen & Yang, 2015; Puram *et al.*, 2016; Roenneberg & Merrow, 2016; Sahar & Sassone-Corsi, 2009; Takahashi *et al.*, 2008). Circadian rhythms are daily biological rhythms synchronized by light and dark cycles of the day/night continuum. Underlying circadian rhythms are oscillations of gene expression occurring in nearly all tissues and cells observed to date ( Koike *et al.*, 2012; Lamia *et al.*, 2008; Lande-Diner *et al.*, 2015; Zhang *et al.*, 2014). Daily cycles of gene transcription and translation are driven by circadian clocks ( Gustafson & Partch, 2015; Mendoza-Viveros *et al.*, 2017; Takahashi, 2017).

An essential component of circadian clocks is the CLOCK-BMAL1 heterodimeric transcription factor ( Bunger *et al.*, 2000; Gekakis *et al.*,1998; Hogenesch *et al.*, 1998; Huang *et al.*, 2012; Lande-Diner *et al.*, 2013; Tamayo *et al.*, 2015). CLOCK-BMAL1 drives the expression of many proteins, including its own repressors, forming the basis of a negative feedback loop ( Kume *et al.*, 1999; Sato *et al.*, 2006). The oscillating abundance of transcriptional repressors leads to daily cycles of CLOCK-BMAL1 target gene expression.

Rhythmic CLOCK-BMAL1 binding to target genes is likely critical to the generation of circadian rhythms ( Koike *et al.*, 2012; Rey *et al.*, 2011; Ripperger & Schibler, 2006). We have previously demonstrated that immobilized DNA oligonucleotides containing E-box DNA binding motifs (CACGTG) can be used to capture native CLOCK-BMAL1, as measured by mass spectrometry ( Tamayo *et al.*, 2015). Here we present a sensitive and versatile method to measure native CLOCK-BMAL1 DNA and chromatin binding from virtually any tissue or cell source in a fast and efficient manner.

## Materials and methods

### Animals

The mouse strain C57/BL6J (The Jackson Laboratory) was used as wildtype (WT), unless the experiment called for a genetically modified animal, in which case an appropriate control animal was utilized. *Bmal1 ^−/−^* animals were bred from heterozygotes in our facility, therefore WT animals were homozygous *Bmal1 ^+/+^* littermates (C57/BL6J background). WT controls for Per2-FH animals were mixed C57BL/6J × 129 genetic background (The Jackson Laboratory). *Bmal1 ^−/−^ mice* ( Bunger *et al.*, 2000) and Per2-FH mice (generated by the Weitz Laboratory of Harvard Medical School) have been previously described ( Duong *et al.*, 2011). Mice were entrained to a 12:12 hr light-dark cycle for at least 2 weeks and then were kept in constant darkness for 24 hrs before sacrificing at the indicated circadian time (CT). CT0 corresponds to the time the lights would turn on, CT4 to 4 hours after that point, and so forth. Mice were euthanized under infrared light, and tissues were dissected under room light. Studies were performed in accordance with the protocol approved by the Harvard Medical School Standing Committee on Animals (protocol #03376).

### Tissue extracts

Tissue and lysate were kept on ice or at 4°C through all steps. As a first step to each tissue extraction, liver was finely minced using a razor, washed with 40 ml of PBS (phosphate buffered saline) per gram of tissue and centrifuged (400 × g, 5 min) until wash solution was clear (8-10×).

Nuclear extracts were prepared as previously described ( Kim *et al.*, 2014), with some amendments. Briefly, tissue was Dounce homogenized with pestle A in Hypotonic Lysis Buffer (250 mM Sucrose, 10 mM HEPES, pH 7.6, protease and phosphatase inhibitors) in a volume 4 times the weight of tissue (e.g. 4 ml/1 g), then centrifuged at 400 × g for 5 min. The pellet was resuspended with 8 ml/1 gr Dounce Buffer (3 ml PBS + 5ml Homogenization Buffer: 2.2 M sucrose, 15 mM KCl, 2 mM EDTA, 10 mM HEPES, pH 7.6), then diluted with 22 ml Homogenization Buffer (assuming 1 gr tissue) and Dounce homogenized with pestle B, followed by ultracentrifugation through a 10 ml sucrose cushion (2.05 M sucrose, 15 mM KCl, 2 mM EDTA, 10 mM HEPES, pH 7.6) at (84,000 × g for 1 hr at 4°C to isolate nuclei (pellet). Nuclei were lysed in nuclear lysis buffer (10 mM Tris-HCl, 300 mM NaCl, 1.5 mM MgCl _2_, 1 mM EDTA, 0.2% TX-100, pH 7.4) containing protease inhibitor cocktail without EDTA (Roche) and phosphatase inhibitor cocktails 2/3 (Sigma-Aldrich). Upon a final centrifugation (20,000 × g, 30 min), the remaining supernatant was liver nuclear extract. For whole tissue extract, the tissue pellet was weighed and resuspended in whole cell extract buffer (10 mM Tris-HCl, 300 mM NaCl, 1.5 mM MgCl _2_, 1 mM EDTA, 0.5% TX-100 [Sigma-Aldrich], pH 7.4, protease and phosphatase inhibitors) in a volume 4× the weight of tissue (e.g. 4 ml/1 g) then Dounce homogenized with pestle A (8 strokes). The homogenate was incubated on ice for 30 min, then centrifuged (20,000 × g, 30 min). The remaining supernatant was whole cell extract. Cytoplasmic extracts were prepared as previously described ( Song *et al.*, 2006) and as also appears elsewhere ( Aryal *et al.*, 2017), with some amendments. Tissue pellet was weighed and resuspended in Hypotonic Lysis Buffer in a volume 4 times the weight of tissue (e.g. 4 ml/1 g), then Dounce homogenized with pestle A (8 strokes). The resulting homogenate was then subjected to a series of centrifugation steps whereupon only the supernatant was retained. Step 1, 1,000 × g, 10min. Step 2, 2,000 × g, 15 min. Step 3, 8,000 × g, 5 min. Step 4, 20,000 × g, 30 min. The supernatant remaining after the final centrifugation was the cytoplasmic extract.

### Cell extracts

To prepare whole cell extracts, PBS washed cells were resuspended in whole cell extract buffer (10 mM Tris-HCl, 300 mM NaCl, 1.5 mM MgCl _2_, 1 mM EDTA, 0.5% TX-100, pH 7.4, protease and phosphatase inhibitors) in a volume 4× the weight of tissue (e.g. 4 ml/1 g), incubated on ice for 30 min, then centrifuged at 20,000 × g for 30 min at 4°C. The remaining supernatant was whole cell extract. 10 µl of whole cell extract diluted in 100 µl final volume of whole cell extract buffer were used for the CPDBA.

### Polyacrylamide gel electrophoresis (PAGE) and immunoblotting

SDS-PAGE/immunoblotting was performed using standard methods. NuPAGE 4–12% polyacrylamide gels (Life Technologies) and NuPAGE LDS sample buffer (with β-mercaptoethanol) were used for SDS-PAGE, and proteins were wet-transferred using a Bio-Rad system (20 mM Tris-HCl, 150 mM glycine, 20% methanol, 0.02% SDS) to PVDF membranes (Millipore) for immunoblotting. 5% skim milk (Millipore) in TBS-T (Tris-HCl buffered saline, 0.01% tween-20) was used as a blocking agent, and TBS-T was used in all washing steps. ECL Prime (GE) was used as an HRP chemiluminescent detection substrate, followed by exposure to film (Denville). CN (clear native) PAGE for the detection of mononucleosomes was performed similarly to previously described BN (blue native) PAGE, except Coomasie blue G dye was omitted from all buffers ( Kim *et al.*, 2014). The sample and 1Kb Plus DNA Ladder (NEB) were mixed with loading dye to a final concentration of 0.17 mg/ml Orange G dye (TCI) and 5% glycerol in 10 mM Bis-Tris (pH 7.0). Samples and DNA ladder were separated on Native PAGE 4–16% Bis-Tris gel (Life Technologies) in 1× anode buffer (50 mM Bis-Tris/HCl [pH 7.0]) and 1× cathode buffer (50 mM Tricine [pH 7.0], 15 mM Bis-Tris) at 4°C.

### Antibodies

The following antibodies were used:

– anti-CLOCK (Abcam, cat# ab3517),
– anti-BMAL1 (generated by the Weitz Laboratory of Harvard Medical School against TDKDDPHGRLEYAEHQGRC and previously described in Tamayo *et al.*, 2015),
– anti-PER2 (ADI, cat# PER21-A),
– anti-CRY1 (Abcam, cat# ab54649),
– anti-HISTONE3 (Abcam, cat# ab1791),
– **Amersham ECL Rabbit IgG, HRP-linked F(ab') _2_ fragment (from donkey)** (GE, cat# NA9340).

All antibodies were polyclonal and raised in rabbits.

### DNA-binding oligonucleotide design

Two oligonucleotide designs were used:

1) Binding/quantitation oligonucleotides,

2) Mononucleosome assembly oligonucleotides.

Each design had two forms: E-box DNA, and control DNA. E-box DNA-binding/quantitation oligonucleotides contained three known CLOCK-BMAL1 binding sites from the Per1 locus, consisting of a canonical E-box sequence (CACGTG) and 10 bp of flanking sequence ( Gekakis *et al.*, 1998). E-box DNA-mononucleosome assembly oligonucleotides contained two copies of each E-box binding site (total of 6 E-box sequences) for a total length of 166bp. Mononucleosome formation requires a minimum of 145bp ( Luger *et al.*, 1997). Control DNA forms were identical to E-box DNA, except that the E-box sequences were scrambled (GCCTGA). All oligonucleotides contained three restriction enzyme sites (SmaI, XhoI, and HpaI) near the 5’ end for native protein elution. The sense strand of each oligonucleotide pair was labeled with a single biotin moiety at the 5’ end. All oligonucleotide sequences are listed below.

### DNA affinity binding

DNA binding of native clock proteins was performed as previously described ( Tamayo *et al.*, 2015) with some amendments. Briefly, sense and anti-sense strands of ssDNA binding/quantitation oligonucleotides were combined (1 µM final) and heated to 94°C for 10 min in high salt annealing buffer (10 mM Tris-HCl, 300 mM NaCl, 2.5 mM MgCl _2_, 0.05% tween-20), then allowed to cool for 1 hr at 25°C to form dsDNA. 150 µl of dsDNA was incubated with 50 µl Dynabeads M-270 Streptavidin (Life Technologies) for 30 min at room temperature (RT). Unbound DNA was washed away with nuclear lysis buffer or cyto lysis buffer. 50 µl of immobilized DNA was incubated with 150 µl of tissue extract (nuclear or cytoplasmic), and incubated for 1 hr at 4°C. Beads were then washed 3× with nuclear lysis buffer prior to elution by 50 µl LDS sample buffer at 98°C for 5 min.

### Clock protein-DNA binding assay (CPDBA)

In PCR tubes (Axygen), 10 µl of Dynabeads M-270 Streptavidin (Life Technologies) were incubated with 100 µl of a concentration range between 1 nM and 100 nM dsDNA binding/quantitation oligonucleotide for 15 min at RT, then washed 3× with high salt annealing buffer. Beads were incubated with ∼8 µg protein or 6–10 µl (∼1 µg/µl) at a final concentration of ∼80 ng/µl (unless a range of extract concentrations is specified) in a final volume of 100 µl nuclear lysis buffer for 30 min at 4°C, then washed 3× with nuclear lysis buffer. Bead-DNA-clock protein conjugates were then incubated with primary antibody (anti-CLOCK or anti-BMAL1) at 1:1000 dilution in TBS-T for 10 min at RT, washed 3× with TBS-T, then incubated with anti-Rabbit IgG (HRP linked) at 1:1000 for 10 min at RT, then washed 3× with TBS-T. The equivalent of 2.5 µl of beads (unless a range is specified) was diluted into 50 µl (final volume) TBS-T in a black/clear bottom 96-well plate (Greiner). 50 µl of ECL Prime chemiluminescent substrate (GE) was added to the well. Data was collected using a Victor3V multi-label reader (Perkin-Elmer) with a 425/60 nm filter. Data analysis and figure preparation were performed using Excel and PowerPoint 2013 (Microsoft).

### Immunoprecipitation of PER2-FH

Immunoprecipitation of PER2-FH and associated proteins was performed as previously described ( Kim *et al.*, 2014). Briefly, nuclear extracts from livers of Per2-FH mice were incubated with FLAG-M2 agarose beads (Sigma-Aldrich) for 2 hr at 4°C. Beads were washed four times with Buffer C (50 mM Tris-HCl, 250 mM NaCl, 1.5 mM MgCl _2_, and 0.2% TX-100, pH 7.5). PER2-FH complexes were eluted with 100 μg/ml FLAG peptide (Sigma-Aldrich) in Buffer C for 30 min at 4°C.

### Phosphatase treatment of extracts

1.7 µl of 800,000 units/mg λ-phosphatase (NEB) were combined with every 6 µl of nuclear extract and incubated in 100 µl of NEBuffer for PMP (50 mM HEPES, 100 mM NaCl, 2 mM DTT, 0.01% Brij 35, pH 7.5) supplemented with 1 mM MnCl _2_, for 45 min at 30°C. This reaction was then analyzed with the CPDBA.

### Cell culture

Mouse hepatoma cells Hep-1c1c7 (ATCC, CRL-2026) were chosen because they are a mouse cell line derived from liver tissue and have been shown to possess functional circadian clocks ( Tong *et al.*, 2010; Yin *et al.*, 2010). Cells were grown in DMEM (Gibco, 1 g/L glucose, L-glutamine, 110 mg/L sodium pyruvate) supplemented with 10% heat inactivated FBS (Atlas), penicillin/streptomycin (Corning) and MEM nonessential amino acids (Cellgro) at 37°C. Cells were passaged using Trypsin/EDTA (Corning).

### Pharmacological inhibition of CKIϵ/δ

Hep-1c1c7 cells were allowed to grow for an additional 48 hrs after they reached 90% confluency prior to treatment. Cell density was an important determinant for successful CPDBA. Cells were incubated with 10 μM of PF670462 or equal volume of DMSO (vehicle) for 48 hrs.

### Mononucleosome reconstitution and CPDBA

PAGE purified ssDNA mononucleosome assembly oligonucleotides (IDT) were annealed by incubation in high salt annealing buffer (10 mM Tris-HCl, 300 mM NaCl, 2.5 mM MgCl _2_, 0.05% Tween-20) for at least 1 hr at RT. The salt concentration of the annealed product was diluted to 150 mM NaCl using low salt annealing buffer (10 mM Tris-HCl, 50 mM NaCl, 1.5 mM MgCl _2_, 0.05% Tween-20) and run on a 2.5% agarose (TBE) gel. The gel between 100 bp and 200 bp was excised, and DNA was extracted using the QIAEX II Agarose gel extraction protocol (Qiagen). The concentration and quality of the resulting dsDNA mononucleosome assembly oligonucleotides were estimated by a spectrophotometer (NanoDrop). The Chromatin Assembly Kit (Active Motif) was used to form mononucleosomes with a few modifications to the protocol. Concentrations of chaperones (hNAP-1 and ACF complex) and HeLa core histones were doubled, and the final incubation was performed for 30 min at 37°C. Mononucleosome formation was observed by running samples on 4–16% native PAGE Bis-Tris gels (Life Technologies) using CN PAGE conditions, as described above, and probing for DNA by incubating the gel in SYBR Gold Nucleic Acid Stain (Thermo Fisher Scientific). To immobilize mononucleosomes, 20 μl of the mononucleosome assembly reaction were incubated with 25 μl of Dynabeads M270 (Life Technologies) for 45 min at 4°C. Beads bound to mononucleosomes were washed 1× with high salt buffer (10 mM Tris pH7.4, 300 mM NaCl, 1.5 mM MgCl2, 0.1% Igepal-CA 630 [Sigma-Aldrich]) and 2× with low salt annealing buffer. Varying concentrations of liver nuclear extracts were incubated with immobilized mononucleosomes for 30 min at RT. Beads were washed 1× with high salt buffer and 2× with wash buffer (10 mM Tris-HCl, 150 mM NaCl, 1.5 mM MgCl _2_, 0.1% TX-100). Beads were analyzed by the CPDBA as described above, or proteins were eluted with LDS sample buffer as described above for SDS-PAGE/immunoblotting.

### Oligonucleotides

E-box DNA-binding/quantitation oligonucleotide sense:

Biotin5’AGTAGTGTTAACCCCGGGCTCGAGCAGTATTTAGCCACGTGACAGTGTAAGCACACGTGGGCCCTCAAGTCCACGTGCAGGGA3’

E-box DNA-binding/quantitation oligonucleotide anti-sense:

5’TCCCTGCACGTGGACTTGAGGGCCCACGTGTGCTTACACTGTCACGTGGCTAAATACTGCTCGAGCCCGGGGTTAACACTACT3’

Control DNA-binding/quantitation oligonucleotide sense:

Biotin5’AGTAGTGTTAACCCCGGGCTCGAGCAGTATTTAGCCTGAGCACAGTGTAAGCACTGAGCGGCCCTCAAGTCCTGAGCCAGGGA3’

Control DNA-binding/quantitation oligonucleotide anti-sense:

5’TCCCTGGCTCAGGACTTGAGGGCCGCTCAGTGCTTACACTGTGCTCAGGCTAAATACTGCTCGAGCCCGGGGTTAACACTACT3’

E-box DNA-mononucleosome assembly sense:

Biotin5’AGTAGTGTTAACCCCGGGCTCGAGCAGTATTTAGCCACGTGACAGTGTAAGCACACGTGGGCCCTCAAGTCCACGTGCAGGGAAGTAGTGTTAACCCCGGGCTCGAGCAGTATTTAGCCACGTGACAGTGTAAGCACACGTGGGCCCTCAAGTCCACGTGCAGGGA3’

E-box DNA-mononucleosome assembly anti-sense:

5’TCCCTGCACGTGGACTTGAGGGCCCACGTGTGCTTACACTGTCACGTGGCTAAATACTGCTCGAGCCCGGGGTTAACACTACTTCCCTGCACGTGGACTTGAGGGCCCACGTGTGCTTACACTGTCACGTGGCTAAATACTGCTCGAGCCCGGGGTTAACACTACT3’

Control DNA-mononucleosome assembly sense:

Biotin5’AGTAGTGTTAACCCCGGGCTCGAGCAGTATTTAGCCTGAGCACAGTGTAAGCACTGAGCGGCCCTCAAGTCCTGAGCCAGGGAAGTAGTGTTAACCCCGGGCTCGAGCAGTATTTAGCCTGAGCACAGTGTAAGCACTGAGCGGCCCTCAAGTCCTGAGCCAGGGA3’

Control DNA-mononucleosome assembly anti-sense:

5’TCCCTGGCTCAGGACTTGAGGGCCGCTCAGTGCTTACACTGTGCTCAGGCTAAATACTGCTCGAGCCCGGGGTTAACACTACTTCCCTGGCTCAGGACTTGAGGGCCGCTCAGTGCTTACACTGTGCTCAGGCTAAATACTGCTCGAGCCCGGGGTTAACACTACT3’

## Results

### Relative quantitation and specificity of CLOCK-BMAL1 DNA binding by the CPDBA

**Figure 1.**
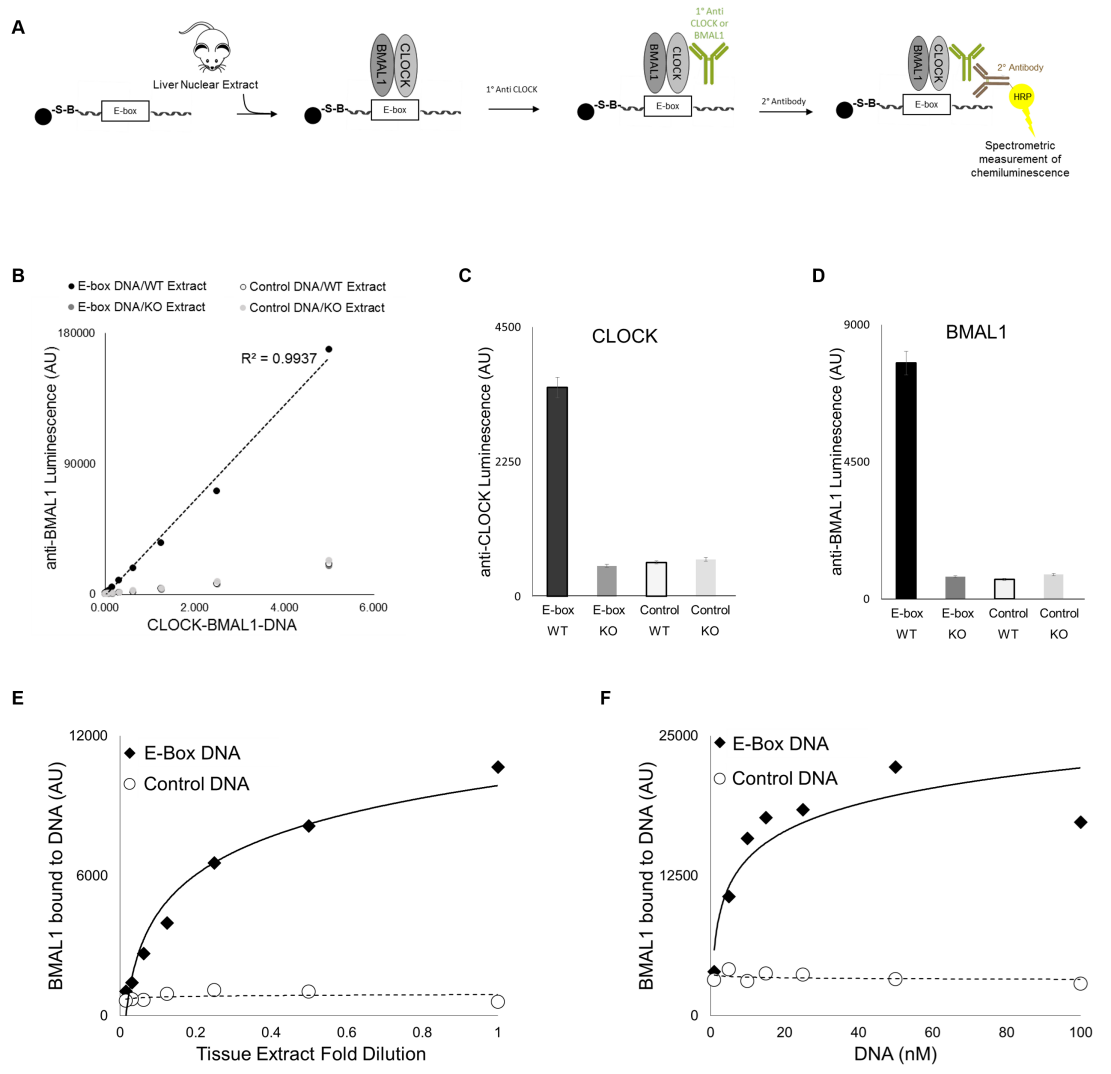
Relative quantitation and specificity of CLOCK-BMAL1 DNA binding by CPDBA. ( **A**) CPDBA design. E-box DNA (or E-box scrambled Control DNA) is immobilized onto a bead substrate. Immobilized DNA is incubated with cell or tissue extract, washed, incubated with primary antibody against CLOCK or BMAL1, washed, incubated with secondary antibody (HRP linked), then washed a final time. The immobilized antibody-protein-DNA complex is then incubated with chemiluminescent substrate (ECL), and analyzed by spectrophotometry (luminescence at 425/60 nm). Counts are arbitrary units. ( **B**) CPDBA was applied to WT nuclear extracts or *Bmal1* ^−/−^ nuclear extracts (KO). The x-axis represents the amount of immobilized antibody-protein-DNA complex (in µl of magnetic beads), used in the final step of the CPDBA, and in the case of WT extract incubated with E-box DNA, corresponds to CLOCK-BMAL1-DNA probed withanti-BMAL1. This experiment was repeated (n=3) with a single volume of immobilized antibody-protein-DNA complex using anti-CLOCK ( **C**) or anti-BMAL1 ( **D**). BMAL1 binding to immobilized DNA was measured from a series of WT nuclear extract dilutions (1 = 16 µg of extract or 160 ng/µl final extract concentration with magnetic beads) while keeping the DNA concentration constant ( **E**), or a series of DNA concentrations were used while keeping the extract concentration constant ( **F**).

We have developed a method to quantitate native CLOCK-BMAL1 DNA binding on immobilized E-box DNA, termed the clock protein- DNA binding assay, or CPDBA ( Figure 1A). 10 µl of nuclear extract from wildtype mouse liver (WT extract) was incubated with immobilized E-box DNA or scrambled E-box DNA (Control DNA) at a single concentration. As an additional control for specificity, we performed parallel experiments using nuclear extracts harvested from *Bmal1* ^−/−^ knockout animals (BKO Extract). Upon extract incubation and anti-BMAL1/secondary antibody incubation with immobilized DNA, varying amounts of immobilized-DNA-protein-antibody (or CLOCK-BMAL1-DNA in the case of WT extract incubated with E-box DNA) were incubated with HRP chemiluminescent substrate, and luminescence data was collected. The presence of BMAL1, as detected by HRP chemiluminescence, was shown to be virtually linear within a given range of CLOCK-BMAL1-DNA, and significantly greater than all control signals ( Figure 1B/CPDBA data). This experiment was repeated with a single concentration of CLOCK-BMAL1-DNA using anti-CLOCK ( Figure 1C/CPDBA data) and anti-BMAL1 for detection ( Figure 1D/CPDBA data), demonstrating a ~5 fold and ~10 fold signal increase over control conditions, respectively. The control conditions were nearly identical to each other, indicating that little or no CLOCK binds to E-box DNA in the absence of BMAL1 *in vitro*. In addition, BMAL1 quantitation showed a dose-dependent relationship with both tissue extract ( Figure 1E/CPDBA data) and DNA concentration ( Figure 1F/CPDBA data).

As a quality control, we performed similar DNA binding experiments using SDS-PAGE/Immunoblotting to qualitatively assess clock protein binding to E-box DNA (see Supplementary Material). We observed that very little CLOCK and BMAL1 were bound to PER2 from cytoplasmic extracts (C) as compared to nuclear extracts (N), as shown by Anti-FLAG co-immunoprecipitation experiments from extracts containing PER2-FLAG-HA ( Figure S1A/Uncropped Figure S1A-B). Since PER2 and CRY1 are known to bind E-box DNA through their interactions with CLOCK-BMAL1 *,* this observation allowed us to use cytoplasmic extracts as an additional negative control for clock protein DNA binding *in vitro.* Nuclear or cytoplasmic extracts were incubated with immobilized E-box DNA or Control DNA (scrambled E-box), and bound proteins were analyzed by SDS-PAGE/immunoblotting for PER2, CRY1, CLOCK and BMAL1. Nuclear but not cytoplasmic PER2, CRY1, CLOCK and BMAL1 bound to E-box DNA ( Figure S1B/Uncropped Figure S1C), further demonstrating specific clock protein interactions with E-box DNA *in vitro*. Nuclear and cytoplasmic markers were distributed as expected ( Figure S2/Uncropped Figure S2). While these results do not preclude the existence of BMAL1 in the cytoplasm, as previously reported by Kwon *et al.*, 2006; Lipton *et al.*, 2015, they suggest that BMAL1 is a predominately nuclear protein.

Taken together, these experiments demonstrate that native CLOCK-BMAL1 DNA binding can be relatively quantitated using tissue extracts as a source of protein and naked DNA as a binding substrate.

### Rhythmic CLOCK-BMAL1 DNA binding measured by CPDBA

Several studies have demonstrated the circadian rhythmicity of CLOCK-BMAL1 E-box DNA binding *in vivo*, as observed by chromatin immunoprecipitation (ChIP) ( Duong *et al.*, 2011; Koike *et al.*, 2012; Ripperger & Schibler, 2006). We asked if CLOCK-BMAL1 DNA binding activity would also oscillate when measured by the CPDBA.

**Figure 2.**
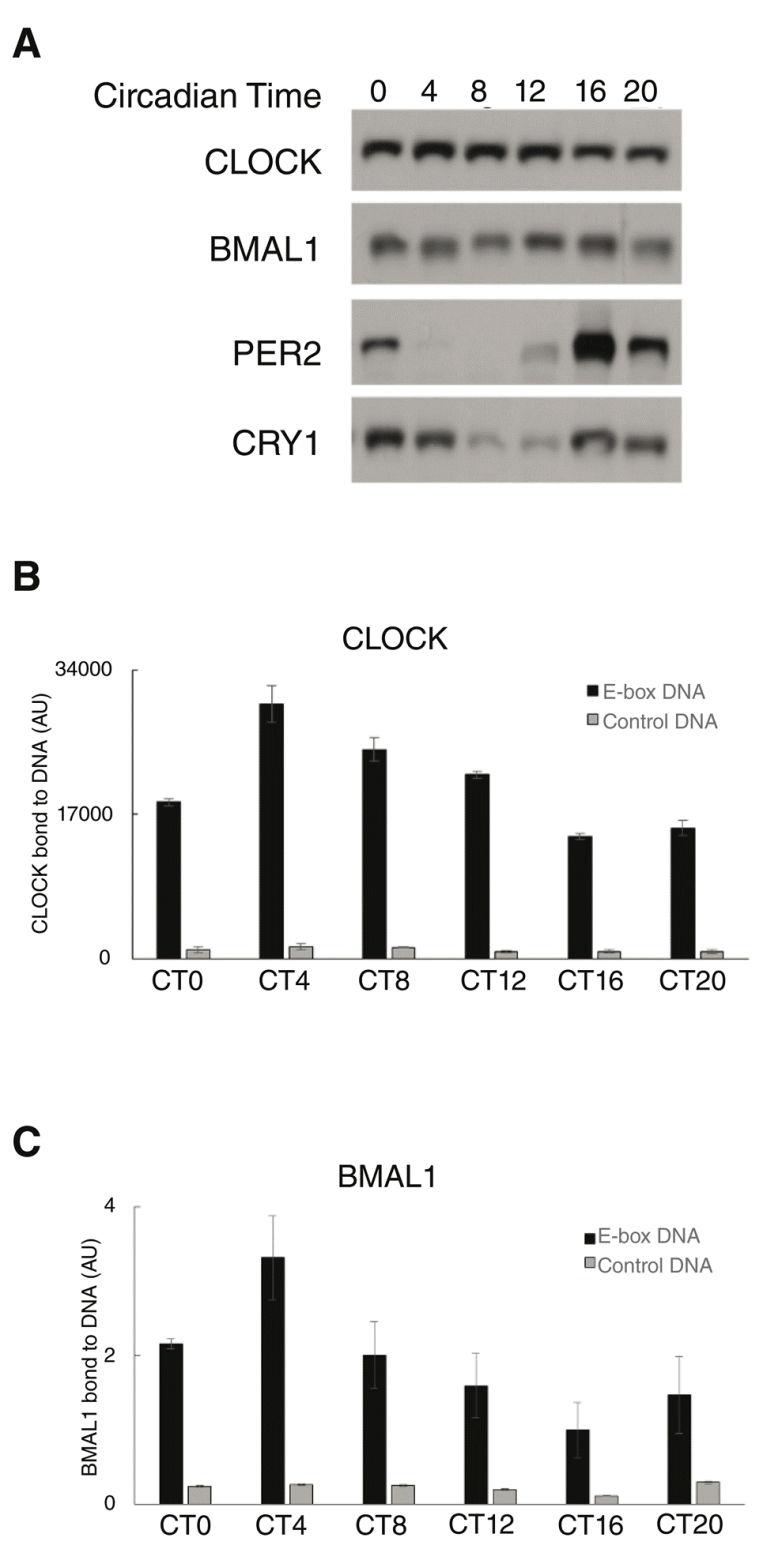
Rhythmic CLOCK-BMAL1 DNA Binding measured by CPDBA. ( **A**) Nuclear extracts were prepared from mouse livers harvested over circadian time (CT). Extracts were analyzed by SDS-PAGE/immunoblotting for the presence of CLOCK, BMAL1, PER2 and CRY1. ( **B**, **C**) CPDBA was applied to these extracts to measure DNA binding by CLOCK ( **B**) or BMAL1 ( **C**) to E-box DNA. Data from technical replicates using extracts from a single mouse are displayed for CLOCK (n=3), and normalized data from multiple mice are displayed for BMAL1 (n=3) The y-axis represents the amount of CLOCK or BMAL1 binding to DNA as measured by the CPDBA (luminescence).

Nuclear extracts were prepared from livers harvested from wildtype mice every 4 hours across circadian time or CT (see methods), and analyzed by SDS-PAGE/immunoblotting for CLOCK, BMAL1, PER2 and CRY1 ( Figure 2A/Uncropped Figure 2). CLOCK and BMAL1 levels were mostly stable across the day, while PER2 and CRY1 levels were highly rhythmic. We then applied the CPDBA to these extracts to monitor CLOCK ( Figure 2B/CPDBA data) and BMAL1 ( Figure 2C/CPDBA data) DNA binding. In both cases, DNA binding reached its peak at CT4 and its trough 12 hours later at CT16, revealing very similar binding patterns to those demonstrated by *in vivo* ChIP experiments. These results show that rhythmic CLOCK-BMAL1 DNA binding activity can be recapitulated using the CPDBA, validating its use as a probe of CLOCK-BMAL1 function.

### CPDBA captures CLOCK-BMAL1 modulation in tissue extracts and cells

**Figure 3.**
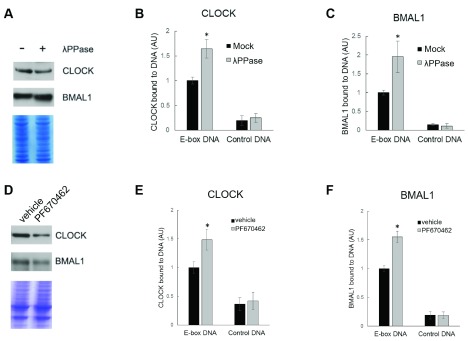
CPDBA captures CLOCK-BMAL1 modulation in tissue extracts and cells. ( **A**) Liver nuclear extracts were treated with lambda phosphatase (λPPase) or mock buffer (Mock). Extracts were analyzed by SDS-PAGE/immunoblotting for the presence of CLOCK and BMAL1, and coomassie stained for loading controls. ( **B**, **C**) CPDBA was applied to these extracts to measure binding of CLOCK ( **B**) or BMAL1 ( **C**) to E-box DNA. All data were normalized to the E-box DNA/mock treated condition (normalized to 1). (* P<0.0001, two-tailed, unequal variance). ( **D**) Extracts were made from Hep-1c1c7 cells incubated with vehicle (DMSO) or 10 µM CKIϵ/δ inhibitor PF670462. Extracts were analyzed by SDS-PAGE/immunoblotting for CLOCK and BMAL1, and coomassie stained for loading controls. ( **E**, **F**) CPDBA was applied to these extracts to measure binding of ( **E**) CLOCK and ( **F**) BMAL1 to E-box DNA. All data were normalized to the E-box DNA/vehicle treated condition (normalized to 1). (*P=<0.0001, two-tailed, unequal variance).

Phosphorylation is the most extensively studied post-translational modification involved in circadian clocks ( Dardente *et al.*, 2007; Kondratov *et al.*, 2006; Lee *et al.*, 2014; Reischl & Kramer, 2011), and has been implicated in the regulation of CLOCK-BMAL1 DNA binding ( Yoshitane *et al.*, 2009; Wang *et al.*, 2013). Nuclear extracts prepared from mouse liver were treated with λ-phosphatase or mock conditions, and analyzed by SDS-PAGE/immunoblotting for CLOCK and BMAL1 ( Figure 3A/Uncropped Figure 3A), indicating similar levels in both conditions. CPDBA was then applied to these extracts to quantitate CLOCK ( Figure 3B/CPDBA data) and BMAL1 ( Figure 3C/CPDBA data) DNA binding to E-box DNA or scrambled E-box DNA (Control DNA). Data across experiments were normalized to the mock treated/E-box DNA bound sample. DNA binding activity increased between 1.5- to 2-fold upon treatment of tissue extract with phosphatase using either antibody.

Pharmacological inhibition of CKIϵ/δ has previously been shown to severely disrupt circadian rhythms ( Isojima *et al.*, 2009; Meng *et al.*, 2010). In this study, mouse hepatoma cells in culture were incubated with a specific kinase inhibitor of CKIϵ/δ (PF670462, IC50 for CKIϵ and CKIδ are 7.7 nM and 14 nM, respectively) or DMSO (vehicle) for 48 hours. This treatment did not discernably affect cell viability. Whole cell extracts were analyzed by SDS-PAGE/immunoblotting for CLOCK and BMAL1 indicating similar or lower levels (as shown) of CLOCK and BMAL1 in the PF670462 treated condition ( Figure 3D/Uncropped Figure 3B). CPDBA was applied to these extracts to quantitate CLOCK ( Figure 3E/CPDBA data) or BMAL1 ( Figure 3F/CPDBA data), and the data across experiments were normalized to the vehicle treated/E-box DNA incubated set. Pharmacological inhibition of CKIϵ/δ resulted in a ~1.5-fold increase of CLOCK-BMAL1 DNA binding activity. Taken together, these results demonstrate the sensitivity and versatility of the CPDBA by capturing less than 2-fold differences in CLOCK-BMAL1 DNA binding activity in a tissue extract and cell culture model of clock modulation.

### Modified CPDBA used to quantitate CLOCK-BMAL1 binding to mononucleosomes

Previous studies have indicated that chromatin modifications alter clock protein access to gene regulatory sites ( Brown *et al.*, 2005; Doi *et al.*, 2006; Duong *et al.*, 2011; Duong & Weitz, 2014; Etchegaray *et al.*, 2003; Kim *et al.*, 2014; Koike *et al.*, 2012; Ripperger & Schibler 2006; Tamayo *et al.*, 2015). Here we asked if native CLOCK-BMAL1 binding to reconstituted nucleosomes can be measured using the CPDBA.

**Figure 4.**
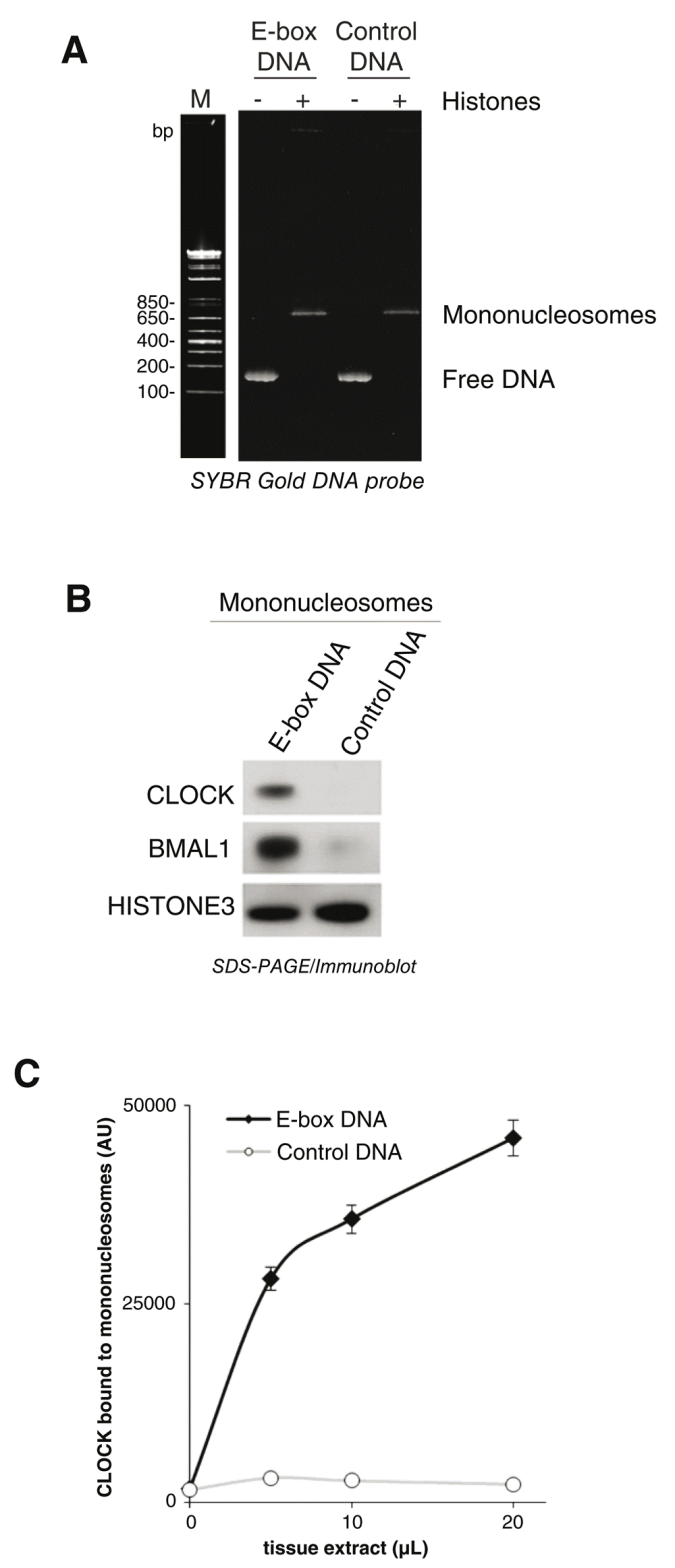
Modified CPDBA used to Quantitate CLOCK-BMAL1 Binding to Mononucleosomes. ( **A**) Free dsDNA containing E-box DNA or scrambled E-box DNA (Control DNA) was incubated with or without core histones, and reconstituted mononucleosomes were analyzed using CN-PAGE stained with SYBR gold. ( **B**) Immobilized mononucleosomes were incubated with liver nuclear extracts. CLOCK-BMAL1 binding to mononucleosomes was assessed using SDS-PAGE/immunoblotting for CLOCK, BMAL1, and HISTONE3. ( **C**) Immobilized mononucleosomes were used in place of naked DNA in CPDBA to measure CLOCK binding to E-box DNA within mononucleosomes using a series of nuclear extract concentrations (n=3).

Mononucleosomes were reconstituted using histone octamers, chromatin assembly chaperones and a biotin tagged 166 base pair oligonucleotide containing E-box sequences or scrambled E-box sequences (Control DNA), then analyzed by CN PAGE/fluorescence DNA labeling ( Figure 4A). Reaction mixtures containing histone octamers shifted the oligonucleotide’s apparent molecular weight to ~600bp, indicating the formation of mononucleosomes ( Figure 4A) ( White *et al.*, 2016). Mononucleosomes containing E-box sequences or scrambled E-box sequences (Control DNA) were immobilized using streptavidin coated magnetic beads, then incubated with nuclear extracts prepared from mouse liver tissue. Bound proteins were analyzed by SDS-PAGE/immunoblotting for CLOCK, BMAL1 and HISTONE3 ( Figure 4B/Uncropped Figure 4), demonstrating that CLOCK-BMAL1 bound specifically to E-box sequences within mononucleosomes *in vitro*. We applied the CPDBA to varying concentrations of nuclear extract using anti-CLOCK ( Figure 4C/CPDBA data), demonstrating a relationship between extract concentration and E-box specific CLOCK binding to mononucleosomes.

Uncropped images of SDS-PAGE/Immunoblots used to construct Figures 2A, 3A, 3D, 4B, S1A, S1B and S2 Click here for additional data file. Copyright: © 2017 Gillessen M et al. 2017 Data associated with the article are available under the terms of the Creative Commons Zero "No rights reserved" data waiver (CC0 1.0 Public domain dedication).

Clock Protein-DNA Binding Assay (CPDBA) data Raw data generated by the CPDBA used to construct Figures 1B–F, 2B, 2C, 3B, 3C, 3E, 3F and 4C Click here for additional data file. Copyright: © 2017 Gillessen M et al. 2017 Data associated with the article are available under the terms of the Creative Commons Zero "No rights reserved" data waiver (CC0 1.0 Public domain dedication).

## Discussion

Previously, we coupled DNA binding selection to quantitative mass spectrometry and discovered novel CLOCK-BMAL1 interacting proteins and chromatin modifying activities ( Tamayo *et al.*, 2015). Here, we present a simple method to measure native CLOCK-BMAL1 DNA and chromatin binding activity from tissue or cell extracts that we term the clock protein-DNA binding assay (CPDBA). Using the CPDBA, we reproduced rhythmic CLOCK-BMAL1 binding from crude tissue extracts in a manner strikingly similar to previously reported chromatin immunoprecipitation (ChIP) patterns ( Duong *et al.*, 2011; Etchegaray *et al.*, 2003; Kim *et al.*, 2014; Koike *et al.*, 2012; Rey *et al.*, 2011; Ripperger & Schibler, 2006; Tamayo *et al.*, 2015). In addition, we show that the CPDBA can be adapted to quantify CLOCK-BMAL1 binding to reconstituted chromatin, in the form of mononucleosomes. This variation of the CPDBA could be used to provide *in vivo* ChIP studies with mechanistic insights. These results indicate that the CPDBA is a viable tool for measuring native CLOCK-BMAL1 DNA binding activity. As such, the CPDBA could complement a variety of research approaches that require monitoring of circadian clock function.

To demonstrate the versatility of the CPDBA, we used it to measure CLOCK-BMAL1 DNA binding activity in both tissue and cell culture extracts, while also using two different approaches to modulate CLOCK-BMAL1 activity. Phosphatase treatment of tissue extracts increased CLOCK-BMAL1 DNA binding as measured by the CPDBA, while treating cells with a specific inhibitor of Casein Kinase ϵ/δ (CKIϵ/δ) yielded similar results. In both cases, the differences between control and experimental conditions were 2-fold or less, demonstrating the sensitivity of the CPDBA. We also performed related experiments using an electrophoretic mobility shift assays (EMSA) to monitor the effect of phosphatase on purified nuclear PER complex (containing CLOCK-BMAL1), and we again detected an increase in DNA binding upon phosphatase treatment ( Aryal *et al.*, 2017). EMSA may surpass the CPDBA in sensitivity and detection limit, since it can use radiolabeled DNA probes to quantify protein-DNA binding. However, the CPDBA is less technically cumbersome and more scalable than EMSAs. We have successfully performed the CPDBA with as little as 6 µl of nuclear extract (~6 µg protein) in 2 hours. While this is already fast and efficient, the CPDBA can likely be improved by further optimization.

The CPDBA is similar to previously reported assays developed for different DNA binding proteins ( Brand *et al.*, 2010; Fischer *et al.*, 2016). While not modeled upon previously described assays, the CPDBA shares features that make it amenable to high throughput approaches, with the potential for automation ( Brand *et al.*, 2013). Furthermore, this method can theoretically be applied to tissue or cells of virtually any source; an important feature given that functional clocks have been observed in most tissues. In conclusion, we submit the CPDBA as a sensitive, fast, efficient and versatile probe of clock function.

## Data availability

The data referenced by this article are under copyright with the following copyright statement: Copyright: © 2017 Gillessen M et al.

Data associated with the article are available under the terms of the Creative Commons Zero "No rights reserved" data waiver (CC0 1.0 Public domain dedication). 

**Dataset 1: Uncropped images of SDS-PAGE/Immunoblots used to construct Figures 2A, 3A, 3D, 4B, S1A, S1B and S2.** DOI, 10.5256/f1000research.11685.d169055 ( Gillessen *et al.*, 2017a)

**Dataset 2: CPDBA data.** Raw data generated by the CPDBA used to construct Figures 1B–F, 2B, 2C, 3B, 3C, 3E, 3F and 4C. DOI, 10.5256/f1000research.11685.d169056 ( Gillessen *et al.*, 2017b)

## Supplementary material

**Figure S1. Native clock proteins bind specifically to immobilized E-box DNA sequences.** Cytoplasmic (C) and nuclear (N) extracts were prepared from the livers of mice expressing PER2-FLAG-HA at CT16. ( **A**) SDS-PAGE/immunoblotting was used to assess the presence of PER2, CLOCK, CRY1 and BMAL1 (equal loading by volume). Anti-FLAG (Sigma-Aldrich) or IgG negative control was used to co-immunoprecipitate clock proteins from C or N extracts. ( **B**) Immobilized dsDNA containing E-box sequences (E-box DNA) or scrambled E-box sequences (Control DNA) were incubated with either C or N extracts. Bound proteins were eluted under denaturing conditions, then analyzed by SDS-PAGE/immuno-blotting for clock proteins.

**Figure S2. Segregation of nuclear and cytoplasmic markers from mouse liver extracts.** Nuclear and cytoplasmic extracts were prepared from mouse livers harvested over circadian time. Extracts were analyzed by SDS-PAGE/immuno-blotting for the presence of RNA polymerase II (nuclear marker) and tubulin (cytoplasmic marker).
