# Supplementary material for: A simple method to measure CLOCK-BMAL1 DNA binding activity in tissue and cell extracts
Source: F1000Res. 2017 Sep 12;6:1316. Originally published 2017 Aug 3. [Version 2] doi: 10.12688/f1000research.11685.2 (PMC5580408; doi:10.12688/f1000research.11685.2)

Uncropped Figure 2. Rhythmic CLOCK-BMAL1 DNA Binding measured by CPDBA. Uncropped images of SDS-PAGE/Immunoblots used to construct Figures 2A.

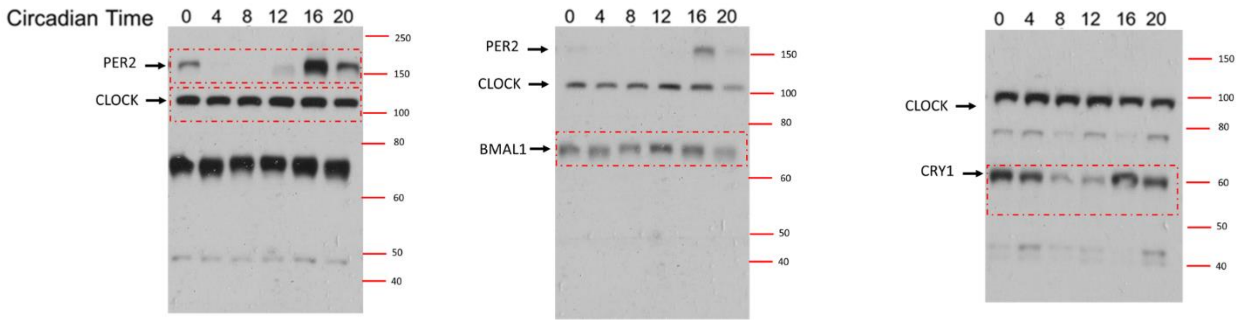

Uncropped Figure 3. CPDBA captures CLOCK-BMAL1 modulation in tissue extracts and cells. Uncropped images of SDS-PAGE/immunoblots used to construct (A) Figure 3A and (B) Figure 3D.

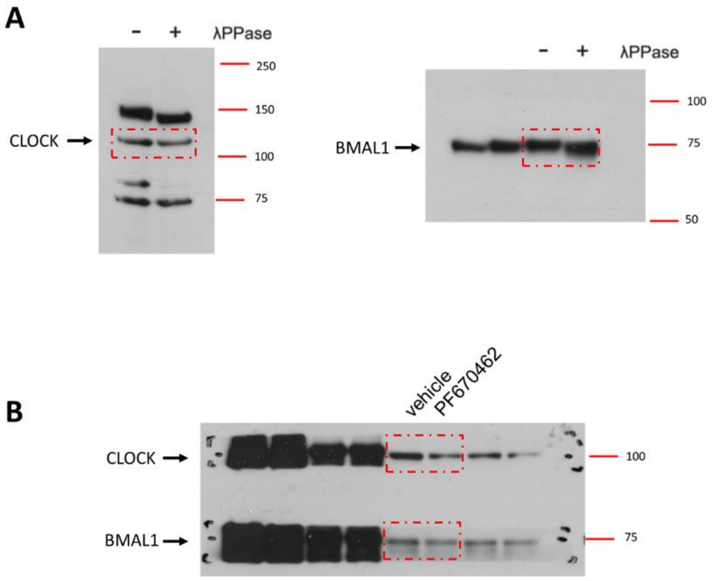

Uncropped Figure 4. Modified CPDBA used to Quantitate CLOCK-BMAL1 Binding to Mononucleosomes. Uncropped images of SDS-PAGE/immunoblots used to construct Figure 4C.

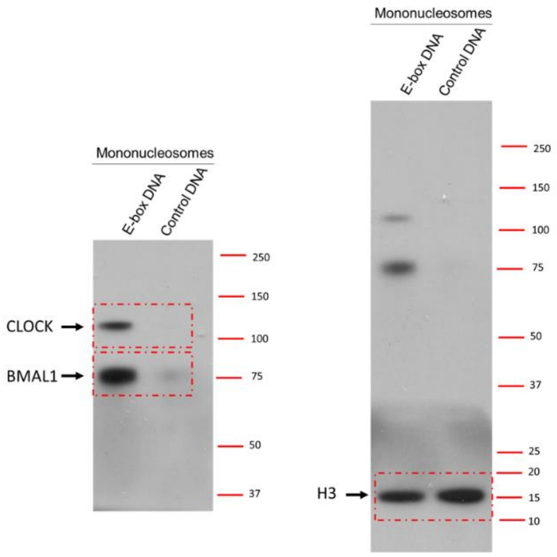

Uncropped Figure S1. Native clock proteins bind specifically to immobilized E-box DNA sequences. Uncropped Images of SDS-PAGE/Immunoblots used to construct Figures S1A and S1B. (A) Input from S1A. (B) DNA affinity binding from S1B. (C) PER-FLAG Immunoprecipitation from S1A.

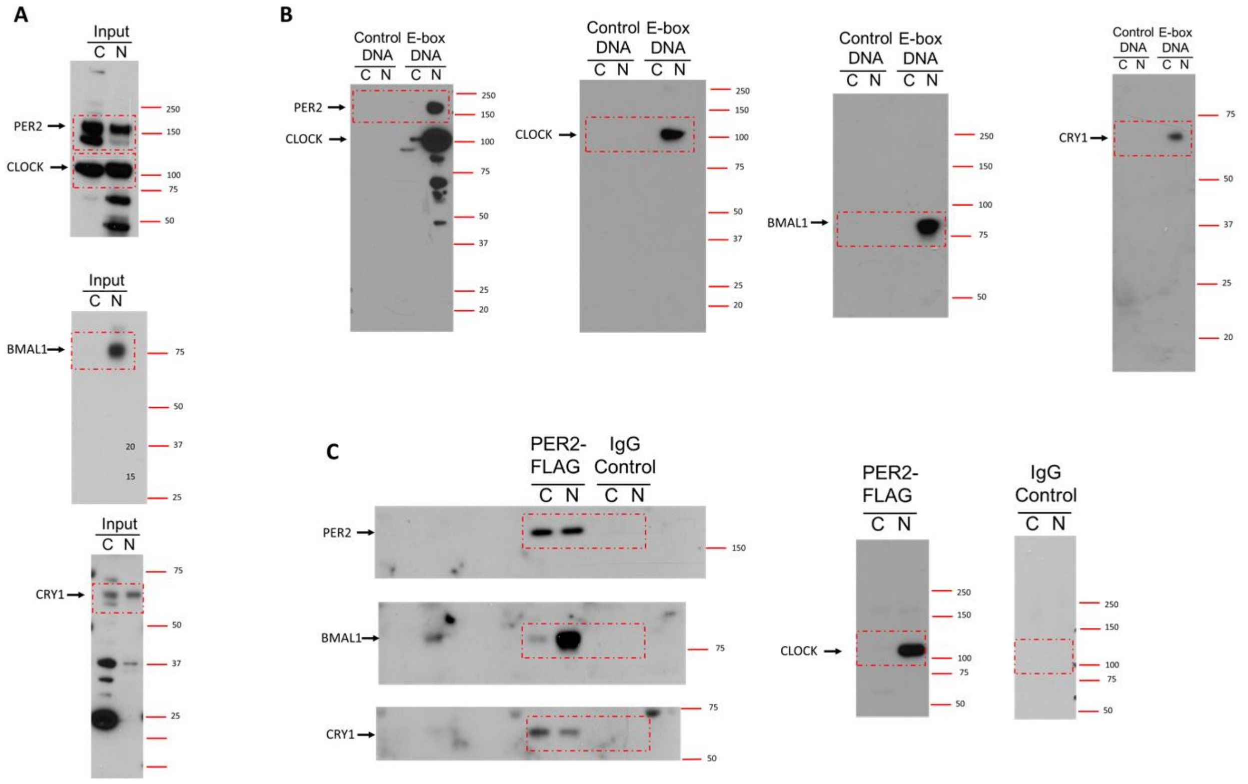

Uncropped Figure S2. Segregation of nuclear and cytoplasmic markers from mouse liver extracts. Uncropped Images of SDS-PAGE/Immunoblots used to construct Figure S2.

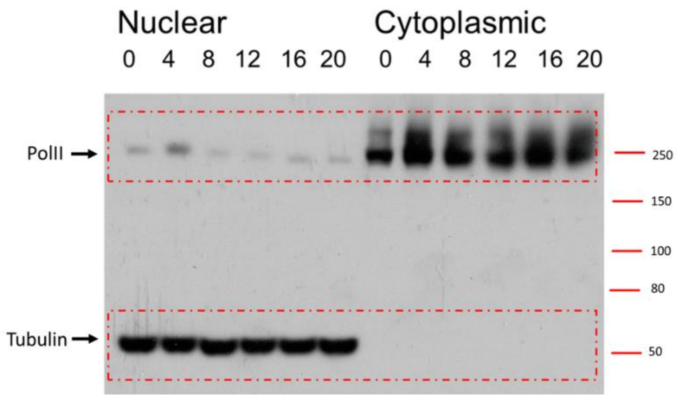

Supplement: Uncropped images of SDS-PAGE/Immunoblots used to construct Figures 2A, 3A, 3D, 4B, S1A, S1B and S2 [file f1000research-6-13702-s0000.tgz › ef7a1bb2-beee-4ce8-9b0e-951c0c9fa476_Uncropped_Figures_-_CL.pdf]
