# Supplementary figures and images for: A simple method to measure CLOCK-BMAL1 DNA binding activity in tissue and cell extracts
Source: F1000Res. 2017 Sep 12;6:1316. Originally published 2017 Aug 3. [Version 2] doi: 10.12688/f1000research.11685.2 (PMC5580408; doi:10.12688/f1000research.11685.2)

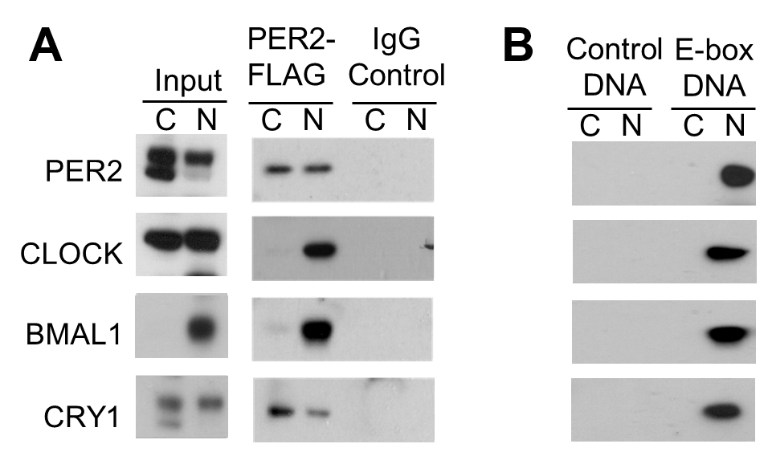

Supplement: Supplementary file 3 [file f1000research-6-13702-s0002.tgz › abb37966-b2cc-422b-b509-4c28e6abbb2f.png]

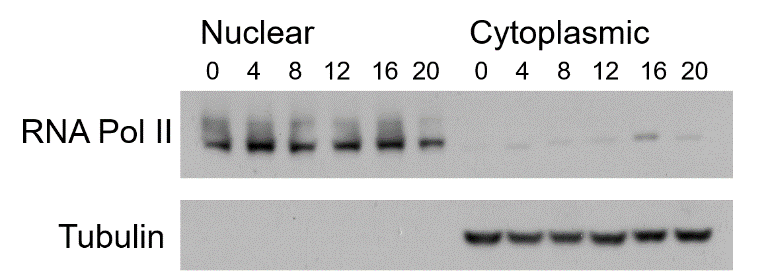

Supplement: Supplementary file 4 [file f1000research-6-13702-s0003.tgz › 2a8a7dfe-f2f9-4dcf-8035-0473c4d8fb58.png]
