# Supplementary material for: A simple method to measure CLOCK-BMAL1 DNA binding activity in tissue and cell extracts
Source: F1000Res. 2017 Sep 12;6:1316. Originally published 2017 Aug 3. [Version 2] doi: 10.12688/f1000research.11685.2 (PMC5580408; doi:10.12688/f1000research.11685.2)
Supplement: Peer review history

# Peer review: Referee response for version 2

**DOI:** 10.5256/f1000research.13702.r25946

**Published:** 2017-09-19

**Authors:** Katja A. Lamia

**Affiliations:** 1 Department of Molecular Medicine, Scripps Research Institute, La Jolla, CA, USA

I thank the authors for their corrections and clarifications and approve the revised manuscript which describes a useful novel method for measuring circadian transcription factors' DNA binding activities.

I have read this submission. I believe that I have an appropriate level of expertise to confirm that it is of an acceptable scientific standard.

# Peer review: Referee response for version 2

**DOI:** 10.5256/f1000research.13702.r25947

**Published:** 2017-09-14

**Authors:** Joanna C. Chiu

**Affiliations:** 1 Department of Entomology and Nematology, College of Agricultural and Environmental Sciences, University of California, Davis, Davis, CA, USA

I am satisfied with the authors' revisions.

I have read this submission. I believe that I have an appropriate level of expertise to confirm that it is of an acceptable scientific standard.

# Peer review: Referee response for version 2

**DOI:** 10.5256/f1000research.13702.r25945

**Published:** 2017-09-13

**Authors:** Yoshitaka Fukada, Naohiro Kon

**Affiliations:** 1 Department of Biological Sciences, School of Science, University of Tokyo, Tokyo, Japan; 2 Medical Science Department, Daiichi Sankyo Co., Ltd., Tokyo, Japan

We would agree to the revision by the authors.

We have read this submission. We believe that we have an appropriate level of expertise to confirm that it is of an acceptable scientific standard.

# Peer review: Referee response for version 1

**DOI:** 10.5256/f1000research.12624.r24900

**Published:** 2017-08-23

**Authors:** Joanna C. Chiu

**Affiliations:** 1 Department of Entomology and Nematology, College of Agricultural and Environmental Sciences, University of California, Davis, Davis, CA, USA

Gillessen *et al.* presents a simple assay to measure DNA binding activity of CLOCK-BMAL1 heterodimers, the major transcriptional activators of clock-regulated genes in animals. They show that the Clock Protein-DNA Binding Assay (CPDBA) can recapitulate CLOCK-BMAL1 DNA binding rhythms from tissue nuclear extracts, detect less than 2-fold differences, and can be used as an alternative to approaches including ChIP-qPCR and gel-shift (EMSA). As opposed to ChIP-qPCR and EMSA, CPDBA may be more amenable to automation and/or high throughput screening of mutations or chemicals to modulate CLOCK-BMAL1 DNA binding activity. I applaud them for going one step further by confirming the functionality of their assay in the context of reconstituted chromatin (mononucleosomes). Overall, this is an excellent and efficient tool, and should not be too difficult for chronobiology colleagues to adopt, especially compared to ChIP and EMSA. I, for one, am excited to try it out.

 Specific comments that the authors should address are detailed below. Most of them are minor.

1. The authors emphasize on a number of occasions that crude tissue extracts can serve as input for the CPDBA (e.g. in the Abstract). In most of the experiments, nuclear extracts as opposed to whole cell extracts were used. Obviously CLOCK-BMAL1 DNA binding takes place in the nucleus, and tissue nuclear extracts will likely produce the cleanest results. Have the authors compare the results of CPDBA using whole cell extracts vs. nuclear extracts? Is it necessary to perform fractionation to collect nuclear extracts for CPDBA. If yes, perhaps the authors should include this recommendation.
2. In the Abstract, the authors mention the assay uses less than 10 microliters or less of crude extract. Perhaps they should specify the protein amount instead of volume. They do state in the Discussion that they assume 1 microliter equals roughly 1microgram (for their extract), but maybe they need to clarify that in the Abstract.
3. The CLOCK-centered Introduction is clear, concise, and well-written, highlighting the importance of studying CLOCK-BMAL1 interaction to DNA in the context of mammalian clock. The authors should consider adding a paragraph discussing factors/modifications that are known to affect DNA binding activity of CLOCK-BMAL1.
4. CPDBA uses HRP conjugated secondary antibodies and chemiluminescent detection. Have the authors consider the use of fluorescent-labeled antibodies and multiplexing? Would fluorescent-labeled antibodies be more quantitative than chemiluminescent detection, and present less problems with signal saturation?
5. The mobility shift the authors intend to show in Figure 3A (and 3D) in cell extracts that are untreated and treated with lambda phosphatase is not obvious at all. Are the authors expecting a significant shift in mobility shift? The authors could try using phostag gel to accentuate the shift.
6. In the section for “Oligonucleotides”, the authors need to add “Biotin” to the first and third primers, just as they did for the mononucleosome assembly primers.
7. Can the authors explain the discrepancies in Y-axis values (DNA binding activity) for Figure 2B vs 2C? The difference in CLOCK (Fig. 2B) and BMAL (Fig. 2C) binding can perhaps be explained by the use of different antibodies. But what about the difference between Figure 2C and Figure 1? The values for Figure 2C seems oddly low, given that both figures are using liver nuclear extracts.
8. Since I am interested in the regulation of clock protein function by post-translational modifications such as phosphorylation, I would be interested to see the authors discuss the possibility of using phosphospecific antibodies (instead of the polyclonal antibodies they list) to detect if specific isoforms of CLOCK and BMAL1 bind preferentially to DNA.

I have read this submission. I believe that I have an appropriate level of expertise to confirm that it is of an acceptable scientific standard.

# Response

**Published:** 2017-09-08

**Authors:** Alfred Tamayo

**Affiliations:** Harvard Medical School, USA

Dear Dr. Chiu. A new version of this manuscript has been submitted with the changes indicated below. Also find below answers to your questions and comments.

1.) “The authors emphasize on a number of occasions that crude tissue extracts can serve as input for the CPDBA (e.g. in the Abstract). In most of the experiments, nuclear extracts as opposed to whole cell extracts were used. Obviously CLOCK-BMAL1 DNA binding takes place in the nucleus, and tissue nuclear extracts will likely produce the cleanest results. Have the authors compare the results of CPDBA using whole cell extracts vs. nuclear extracts? Is it necessary to perform fractionation to collect nuclear extracts for CPDBA. If yes, perhaps the authors should include this recommendation.”

Answer: It is not necessary to use nuclear extracts in the CPDBA, whole cell extracts also work. However we have not performed head-to-head comparisons of nuclear extracts versus whole cell extracts, so we cannot recommend one over the other. 

2.) “In the Abstract, the authors mention the assay uses less than 10 microliters or less of crude extract. Perhaps they should specify the protein amount instead of volume. They do state in the Discussion that they assume 1 microliter equals roughly 1microgram (for their extract), but maybe they need to clarify that in the Abstract.”

Answer: A better description of the protein concentrations of extracts used in all assays, unless a concentration range is stated, has been added to Materials and Methods: “Beads were incubated with ~8 µg protein or 6–10 µl (~1 µg/µl) at a final concentration of ~80 ng/µl of extract (unless a range of extract concentrations is specified)…”  In addition, the concentration equivalent to 1 in Figure 1E is now noted in the legend: “1 = 16 µg of extract or 160 ng/µl final extract concentration with magnetic beads.” 

3.) “The CLOCK-centered Introduction is clear, concise, and well-written, highlighting the importance of studying CLOCK-BMAL1 interaction to DNA in the context of mammalian clock. The authors should consider adding a paragraph discussing factors/modifications that are known to affect DNA binding activity of CLOCK-BMAL1.”

Answer: A more detailed discussion of factors affecting DNA binding may not be in keeping with the spirit of a methods article. However, such a discussion is certainly worth having in light of our findings, so we included it in this comment (below), which is also publically available.

The molecular underpinnings governing CLOCK-BMAL1 DNA binding activity are not fully elucidated. Repressor proteins physically interact with CLOCK-BMAL1, suggesting steric or post-translational regulation of CLOCK-BMAL1 activity (Ye *et al.* 2011, Ye *et al.* 2014, Chiou et al. 2016, Michael *et al.* 2017). In addition, a body of work indicates epigenetic regulation of DNA accessibility as an underlying cause (Etchegary *et al.* 2003, Brown *et al*. 2005, Doi *et al*. 2006, Ripperger and Schibler 2006, Duong *et al*. 2011, Koike *et al. *2012).  Direct repression of CLOCK-BMAL1 activity and gating of chromatin accessibility are not mutually exclusive, therefore, how are these modalities integrated?

Phosphorylation dependent restriction of CLOCK-BMAL1 DNA binding has been implicated by previous reports. Mutagenic replacement of serine residues with phospho-mimicking amino acids within the basic helix-loop-helix (bHLH) domains of either CLOCK (S38, S42) or BMAL1 (S78) disrupts DNA binding (Yoshitane *et al. *2009, Wang *et al. *2013). However, CLOCK phosphorylation at S440, S441, and S446 is correlated with CLOCK-BMAL1 activation (Robles *et al.* 2016). These findings point to an intricate coordination of clock protein phosphorylation tied to structure-function relationships, which we may begin to unravel using methods described in this study.

4.) “CPDBA uses HRP conjugated secondary antibodies and chemiluminescent detection. Have the authors consider the use of fluorescent-labeled antibodies and multiplexing? Would fluorescent-labeled antibodies be more quantitative than chemiluminescent detection, and present less problems with signal saturation?”

Answer: Yes we have considered using fluorescent-labeled antibodies as well as other alterations to the original technique such as TR-FRET (time resolved fluorescence energy transfer) and ALPHAScreen technology, however, we haven’t tried these adaptations as yet.

5.) “The mobility shift the authors intend to show in Figure 3A (and 3D) in cell extracts that are untreated and treated with lambda phosphatase is not obvious at all. Are the authors expecting a significant shift in mobility shift? The authors could try using phostag gel to accentuate the shift.”

Answer: In general, upon phosphatase treatment of extract, we did not see a mobility shift for CLOCK, we did see a subtle shift for BMAL1 and a significant shift for PER2 (not shown) using standard SDS-PAGE/immunoblotting. As you point out, a lack of shift does not exclude effective dephosphorylation. Using phostag gels is an excellent suggestion, though we have not attempted. MS analysis of immunoprecipitated proteins from phosphatase treated extracts would also be informative, if less practical. However, a more thorough analysis of the role of phosphorylation in CLOCK-BMAL1 DNA binding is beyond the scope of this manuscript. 

6.) “In the section for “Oligonucleotides”, the authors need to add “Biotin” to the first and third primers, just as they did for the mononucleosome assembly primers.”

Answer: The requested modification has been made to Materials and Methods.

7.) “Can the authors explain the discrepancies in Y-axis values (DNA binding activity) for Figure 2B vs 2C? The difference in CLOCK (Fig. 2B) and BMAL (Fig. 2C) binding can perhaps be explained by the use of different antibodies. But what about the difference between Figure 2C and Figure 1? The values for Figure 2C seems oddly low, given that both figures are using liver nuclear extracts prepared.”

Answer: Figure 2B represents raw counts from a single set of nuclear extracts prepared simultaneously, i.e. one set of mice. While Figure 2C represents independently prepared extracts from several sets of mice. Raw counts across sets of mouse extracts were too variable to be represented in the same graph. Independently prepared extracts were used in Figure 2C and Figure 1, which is why the scales are different. We do not fully understand the reason behind this, but it is an important technical observation.

8.) “Since I am interested in the regulation of clock protein function by post-translational modifications such as phosphorylation, I would be interested to see the authors discuss the possibility of using phosphospecific antibodies (instead of the polyclonal antibodies they list) to detect if specific isoforms of CLOCK and BMAL1 bind preferentially to DNA.”

Answer: This is a very interesting suggestion, as using phospho-specific antibodies in the CPDBA could give an initial indication of differential DNA binding affinities across phosphospecies either across circadian time and/or within a population of molecules at a given CT. Perhaps it would be best if the assay could be multiplexed to use phospho-state and non-phospho specific antibodies simultaneously from the same sample.

Best Regards

# Peer review: Referee response for version 1

**DOI:** 10.5256/f1000research.12624.r24783

**Published:** 2017-08-21

**Authors:** Katja A. Lamia

**Affiliations:** 1 Department of Molecular Medicine, Scripps Research Institute, La Jolla, CA, USA

Gillessen, Kwak, and Tamayo describe a method for measuring the interaction of CLOCK and BMAL1 purified from cellular or tissue extracts with either naked DNA or nucleosomes including an established CLOCK-BMAL1 binding sequence containing several E-boxes. The manuscript is well written, and the method will be broadly useful, especially as it could be adapted to other transcription factors by altering the target DNA sequences. Given that it is a method description, it is important that all details of the protocol are clear and I include here some suggestions for clarification.

 Materials and methods:

1. In the description of the preparation of tissue extracts, the following is insufficiently clear: “…tissue was Dounce homogenized with pestle A, centrifuged, then Dounce homogenized with pestle B, followed by…” Was the supernatant discarded after centrifugation between homogenization with pestle A and pestle B? If so, was the pellet resuspended again in the same buffer?
2. Please provide detailed composition and volumes of buffers used for initial homogenization and sucrose cushion centrifugation.
3. In the list of antibodies for the last item, either delete “ECL” or include the full product name “Amersham ECL Rabbit IgG, HRP-linked F(ab’)2 fragment (from donkey)”. Also, I can’t find a product number with a “V” at the end – is that a typo?
4. Either place the oligonucleotide sequences within the section describing “DNA-binding oligonucleotide design” or include a sentence stating that the sequences are listed below.
5. Nowhere in the manuscript does it state the concentration of extracts that is used in the assays and this is critical information. Perhaps this was meant to be included in the section describing the CPDBA where it states “Beads were incubated with 6-10 l of extract (unless a range of concentrations is specified)…” ?
6. The section titled “Pharmacological inhibition of CKIe/d”also includes details of preparation of cell extracts, which might fit more logically as a separate section following preparation of tissue extracts.

 

 Results :

1. Related to point #5 above, there is no mention of the concentration of extract used in experiments shown in Fig. 1. What does arbitrary units refer to on the x and y axes in Fig. 1B? In Fig. 1E and Fig. 4C, the x axis is labeled “fold dilution” or “l extract” but it would be much more informative to know what concentration of extract is included in the assay.
2. In Figs 3A and 3D, the legend refers to Western blot detection of PER2 and CKId that does not appear in the figure.
3. The text states that there are similar levels of CLOCK and BMAL1 protein in each condition in Fig 3D but the figure shows much less CLOCK and BMAL1 protein in the extract treated with PF671462. Both Fig. 3A and 3D should include a loading control blot as well. Since the change in protein amounts appears to be opposite to the change in binding measured, this does not invalidate the conclusions but the data should be described accurately in the text.

I have read this submission. I believe that I have an appropriate level of expertise to confirm that it is of an acceptable scientific standard, however I have significant reservations, as outlined above.

# Response

**Published:** 2017-09-08

**Authors:** Alfred Tamayo

**Affiliations:** Harvard Medical School, USA

Dear Dr. Lamia. A new version of this manuscript has been submitted with the changes indicated below.

1.)”In the description of the preparation of tissue extracts, the following is insufficiently clear: “…tissue was Dounce homogenized with pestle A, centrifuged, then Dounce homogenized with pestle B, followed by…” Was the supernatant discarded after centrifugation between homogenization with pestle A and pestle B? If so, was the pellet resuspended again in the same buffer?”

Answer: Please see changes made to Materials and Methods: “The pellet was resuspended withn 8 ml/1 gr Dounce Buffer (3 ml PBS + 5ml Homogenization Buffer: 2.2 M sucrose, 15 mM KCl, 2 mM EDTA, 10 mM HEPES, pH 7.6), then diluted with 22 ml Homogenization Buffer (assuming 1 gr tissue)”

2.) “Please provide detailed composition and volumes of buffers used for initial homogenization and sucrose cushion centrifugation.”

Answer: Requested information added to Materials and Methods: “…Hypotonic Lysis Buffer (250 mM Sucrose, 10 mM HEPES, pH 7.6, protease and phosphatase inhibitors) in a volume 4 times the weight of tissue (e.g. 4 ml/1 g), thencentrifuged at 400 × g for 5 min. The pellet was resuspended with 8 ml/1 gr Dounce Buffer (3 ml PBS + 5ml Homogenization Buffer: 2.2 M sucrose, 15 mM KCl, 2 mM EDTA, 10 mM HEPES, pH 7.6), then diluted with 22 ml Homogenization Buffer (assuming 1 gr tissue) and Dounce homogenized with pestle B, followed by ultracentrifugation through a 10 ml sucrose cushion (2.05 M sucrose, 15 mM KCl, 2 mM EDTA, 10 mM HEPES, pH 7.6) at84,000 × g for 1 hr at 4°C to isolate nuclei (pellet)…”

3.)”In the list of antibodies for the last item, either delete “ECL” or include the full product name “Amersham ECL Rabbit IgG, HRP-linked F(ab’)2 fragment (from donkey)”. Also, I can’t find a product number with a “V” at the end – is that a typo?”

Answer: Yes, the typo has been corrected and the requested information modified and added.

4.) “Either place the oligonucleotide sequences within the section describing “DNA-binding oligonucleotide design” or include a sentence stating that the sequences are listed below.”

Answer: Requested sentence has been added.

5.) “Nowhere in the manuscript does it state the concentration of extracts that is used in the assays and this is critical information. Perhaps this was meant to be included in the section describing the CPDBA where it states “Beads were incubated with 6-10 l of extract (unless a range of concentrations is specified)…” ?”

Answer: Changes were made to the Materials and Methods to better indicate the protein concentrations used: “Beads were incubated with ~8 µg protein or 6–10 µl (~1 µg/µl) at a final concentration of ~80 ng/µl of extract (unless a range of extract concentrations is specified)…”

6.) “The section titled “Pharmacological inhibition of CKIe/d”also includes details of preparation of cell extracts, which might fit more logically as a separate section following preparation of tissue extracts.”

Answer: A separate section describing the preparation of cell extracts has been added to Materials and Methods.

7.) “Related to point #5 above, there is no mention of the concentration of extract used in experiments shown in Fig. 1. What does arbitrary units refer to on the x and y axes in Fig. 1B? In Fig. 1E and Fig. 4C, the x axis is labeled “fold dilution” or “l extract” but it would be much more informative to know what concentration of extract is included in the assay.”

Answer: As noted above in the answer to point #5, a better description of the protein concentrations of extracts used in all assays, unless a concentration series is stated, has been added to Materials and Methods. In addition, the concentration equivalent to 1 in Figure 1E is now noted in the legend: “1 = 16 µg of extract or 160 ng/µl final extract concentration with magnetic beads.” In Figure 1B, AU in the x-axis is a typo and has been changed (x-axis in this case is ul of magnetic beads, as noted in the legend). Y-axis AUs are raw luminescence counts from a plate reader.

8.) “In Figs 3A and 3D, the legend refers to Western blot detection of PER2 and CKId that does not appear in the figure.”

Answer: This was a typo and has been corrected.

9.)”The text states that there are similar levels of CLOCK and BMAL1 protein in each condition in Fig 3D but the figure shows much less CLOCK and BMAL1 protein in the extract treated with PF671462. Both Fig. 3A and 3D should include a loading control blot as well. Since the change in protein amounts appears to be opposite to the change in binding measured, this does not invalidate the conclusions but the data should be described accurately in the text.”

Answer: Please see revised Results section addressing the description of the data: “Whole cell extracts were analyzed by SDS-PAGE/immunoblotting for CLOCK and BMAL1 indicating similar or lower levels (as shown) of CLOCK and BMAL1 in the PF670462 treated condition  ( Figure 3D/Uncropped Figure 3B)…” 

Also, to Figures 3A and 3D we have added portions of SDS-PAGE/coomassie stained gels which we believe are equal or better than western blot controls to assess equal loading. 

Best Regards

# Peer review: Referee response for version 1

**DOI:** 10.5256/f1000research.12624.r24786

**Published:** 2017-08-17

**Authors:** Yoshitaka Fukada, Naohiro Kon

**Affiliations:** 1 Department of Biological Sciences, School of Science, University of Tokyo, Tokyo, Japan; 2 Medical Science Department, Daiichi Sankyo Co., Ltd., Tokyo, Japan

In the present work, the authors developed a novel method to detect DNA binding activity of CLOCK-BMAL1 complex with a fixed DNA probe containing an E-Box repeat. The method is based on a simple procedure and could be useful for studies including high-throughput investigation of the regulatory mechanism on CLOCK-BMAL1.

 It is desirable to compare the new method with previously established ones, e.g., EMSA and ChIP-PCR in parallel experiments by using the same samples. Then, readers could easily understand advantage/disadvantage of the present assay, in terms of detection limit, the range, and sensitivity etc.

 The data in Figure 3 demonstrate enhanced DNA-binding activities of CLOCK:BMAL1 by pre-treatment with lambda phosphatase and CKI-epsilon/delta inhibitor (PF670462). We are interested in a potential change of the CLOCK-BMAL1 activity under previously described conditions. For example, we reported several treatments that reduce the transactivation ability of CLOCK-BMAL1 complex: MAPK-dependent phosphorylation of BMAL1, phospho-mimic mutation in CLOCK, reduced CLOCK-BMAL1 dimerization under CaMKII inhibition [1,2,3]

We have read this submission. We believe that we have an appropriate level of expertise to confirm that it is of an acceptable scientific standard.

## References

[1] SanadaKOkanoTFukadaY: Mitogen-activated protein kinase phosphorylates and negatively regulates basic helix-loop-helix-PAS transcription factor BMAL1. *J Biol Chem*.2002;277(1) : 10.1074/jbc.M107850200 267-71 10.1074/jbc.M107850200 11687575

[2] YoshitaneHTakaoTSatomiYDuNHOkanoTFukadaY: Roles of CLOCK phosphorylation in suppression of E-box-dependent transcription. *Mol Cell Biol*.2009;29(13) : 10.1128/MCB.01864-08 3675-86 10.1128/MCB.01864-08 19414601PMC2698759

[3] KonNYoshikawaTHonmaSYamagataYYoshitaneHShimizuKSugiyamaYHaraCKameshitaIHonmaKFukadaY: CaMKII is essential for the cellular clock and coupling between morning and evening behavioral rhythms. *Genes Dev*.2014;28(10) : 10.1101/gad.237511.114 1101-10 10.1101/gad.237511.114 24831701PMC4035538

# Response

**Published:** 2017-09-08

**Authors:** Alfred Tamayo

**Affiliations:** Harvard Medical School, USA

Dear Dr. Fukada and Dr. Kon. A new version of this manuscript has been submitted with the changes indicated below.

1.) “It is desirable to compare the new method with previously established ones, e.g., EMSA and ChIP-PCR in parallel experiments by using the same samples. Then, readers could easily understand advantage/disadvantage of the present assay, in terms of detection limit, the range, and sensitivity etc.”

Answer: While it is beyond the scope of this work to perform parallel experiments with other techniques, we comment on their comparison in our revised Discussion section: “In addition, we show that the CPDBA can be adapted to quantify CLOCK-BMAL1 binding to reconstituted chromatin, in the form of mononucleosomes. This variation of the CPDBA could be used to provide *in vivo* ChIP studies with mechanistic insights.… We also performed related experiments using an electrophoretic mobility shift assays (EMSA) to monitor the effect of phosphatase on purified nuclear PER complex (containing CLOCK-BMAL1), and we again detected an increase in DNA binding upon phosphatase treatment (Aryal and Kwak *et al.*, 2017). EMSA may surpass the CPDBA in sensitivity and detection limit, since it can use radiolabeled DNA probes to quantify protein-DNA binding. However, the CPDBA is less technically cumbersome and more scalable than EMSAs.”

2.) “The data in Figure 3 demonstrate enhanced DNA-binding activities of CLOCK:BMAL1 by pre-treatment with lambda phosphatase and CKI-epsilon/delta inhibitor (PF670462). We are interested in a potential change of the CLOCK-BMAL1 activity under previously described conditions. For example, we reported several treatments that reduce the transactivation ability of CLOCK-BMAL1 complex: MAPK-dependent phosphorylation of BMAL1, phospho-mimic mutation in CLOCK, reduced CLOCK-BMAL1 dimerization under CaMKII inhibition [1,2,3]”

Answer: The complex relationship between phosphorylation and CLOCK-BMAL1 regulation is indeed interesting and requires a comprehensive understanding of the causal relationship between site-specific phosphorylation, biochemical activity and biological consequence. As you point out, it would be interesting to interrogate the roles of other kinases, such as MAPKs and CaMKII in CLOCK-BMAL1 DNA binding using the CPDBA. We should note that dose and exposure time would need to be optimized in any kinase inhibitor study, as was done for PF670462, though we do not mention it in the manuscript. We must also point out however that this assay cannot assess CLOCK-BMAL1 transactivation activity downstream of DNA binding.

Also, we have changed how your work is cited in the Results section to better distinguish it from the other works cited.

Best Regards
